# Hylin-a1: A Pan-Inhibitor against Emerging and Re-Emerging Respiratory Viruses

**DOI:** 10.3390/ijms241813888

**Published:** 2023-09-09

**Authors:** Annalisa Chianese, Carla Zannella, Alessandra Monti, Nunzianna Doti, Giuseppina Sanna, Aldo Manzin, Anna De Filippis, Massimiliano Galdiero

**Affiliations:** 1 Department of Experimental Medicine, University of Campania “Luigi Vanvitelli”, 80138 Naples, Italy; annalisa.chianese@unicampania.it (A.C.); carla.zannella@unicampania.it (C.Z.); anna.defilippis@unicampania.it (A.D.F.); 2 Institute of Biostructures and Bioimaging (IBB), National Research Council (CNR), 80131 Naples, Italy; alessandra.monti@ibb.cnr.it (A.M.); nunzianna.doti@cnr.it (N.D.); 3 Department of Biomedical Sciences, University of Cagliari, Cittadella Universitaria, 09042 Monserrato, Italy; g.sanna@unica.it (G.S.); aldo.manzin@unica.it (A.M.)

**Keywords:** antimicrobial peptide, inhibitory peptide, respiratory viruses, coronavirus, SARS-CoV-2, influenza, emerging infection

## Abstract

Pandemic and epidemic outbreaks of respiratory viruses are a challenge for public health and social care system worldwide, leading to high mortality and morbidity among the human populations. In light of the limited efficacy of current vaccines and antiviral drugs against respiratory viral infections and the emergence and re-emergence of new viruses, novel broad-spectrum antiviral drugs are needed for the prevention and treatment of these infections. Antimicrobial peptides with an antiviral effect, also known as AVPs, have already been reported as potent inhibitors of viral infections by affecting different stages of the virus lifecycle. In the present study, we analyzed the activity of the AVP Hylin-a1, secreted by the frog *Hypsiboas albopunctatus*, against a wide range of respiratory viruses, including the coronaviruses HCoV-229E and SARS-CoV-2, measles virus, human parainfluenza virus type 3, and influenza virus H1N1. We report a significant inhibitory effect on infectivity in all the enveloped viruses, whereas there was a lack of activity against the naked coxsackievirus B3. Considering the enormous therapeutic potential of Hylin-a1, further experiments are required to elucidate its mechanism of action and to increase its stability by modifying the native sequence.

## 1. Introduction

Viral respiratory infections constitute a large public health problem and are estimated as one of the top causes of death in children <5 years old [1,2,3]. Data from the Global Burden of Diseases Study 2019 (GBD 2019) indicated that 3.97 million people died worldwide in 2019, and 9190 were children under 5 years [4]. These numbers greatly increased after the pandemic caused by severe acute respiratory syndrome coronavirus 2 (SARS-CoV-2). Furthermore, the COVID-19 pandemic highly influenced the transmission and epidemiology of other respiratory viruses, especially respiratory syncytial virus (RSV). It is estimated that RSV infection shifted its seasonal peak when the COVID-19 counteracting measures decreased, by increasing, on the other hand, the number of infected people and the severity of disease [4].

Among about 200 viruses responsible of respiratory diseases, the most common are influenza virus and RSV. Other causes of viral respiratory infections include human coronaviruses (HCoVs), human parainfluenza viruses (HPIV), measles virus (MeV), coxsackieviruses, rhinoviruses, metapneumoviruses, and adenoviruses [5,6,7]. Additionally, herpesviruses such as cytomegalovirus, varicella zoster virus, and herpes simplex virus, can also be involved in respiratory illnesses in immunocompromised people [8,9]. The COVID-19 pandemic has clearly highlighted how respiratory viral infections can impact individual and public health as well as cause significant economic and social damage. However, vaccines are only available for the influenza virus, measles virus, and for SARS-CoV-2, whereas for other viruses they are still in development. Given the long timeframe necessary to realize vaccines and the emergence of viral variants that could compromise their use, antiviral drugs could constitute a valid treatment option. However, even today we lack an arsenal of antivirals capable of preventing or treating respiratory diseases. Currently, the only antiviral drugs available are those against influenza viruses and, in some cases, against RSV. However, even for these agents, their effectiveness has been slowed by the emergence of drug resistance. For example, influenza A viruses are resistant to adamantanes [10,11,12,13] and oseltamivir [14,15], the two drugs most frequently used to treat flu. Ribavirin, a nucleoside analogue blocking the replication of many DNA and RNA viruses, is mostly recommended in severe RSV infections in infants [16], but in recent years a growing number of ribavirin-resistant strains have been identified [17]. Therefore, researchers continue to devote efforts to the discovery of broad-spectrum antivirals.

Antimicrobial peptides (AMPs) with antiviral effects (antiviral peptides: AVPs) have already shown great activity against a wide range of viruses by affecting different stages of their lifecycle [18,19,20,21,22,23,24,25,26]; this has led to them becoming alternative pharmaceutically available antiviral drugs. Amphibians are the largest group of animals able to produce natural AVPs. They include more than 8000 species, most of which belong to the order Anura, and new species are discovered each year. The skin represents an essential barrier for these animals by controlling the exchanges between the internal and external environments and, therefore, it is constantly in contact with several environmental factors. It is well known that anurans release through the skin surface peptides with various biological activities ranging from the anti-cancer, anti-inflammatory, and anti-diabetes activities to antimicrobial effects [27,28,29,30,31]. To date, 53 AVPs out of the 201 listed in the Antimicrobial Peptides Database (last access: 6 January 2023) are derived from amphibians.

Esculentins are one of the first families of peptides identified. Currently, they have been obtained from the skin of 13 different anurans [32,33,34,35] and their anti-infective properties are widely reported. In detail, esculentin-1GN (GLFSKKGGKGGKSWIKGVFKGIKGIGKEVGGDVIRTGIEIAACKIKGEC), derived from the frog *Sylvirana guentheri*, has been described as a multifunctional peptide [36,37,38] with anti-influenza A activity [39]. In fact, recently, Yang et al. demonstrated that esculentin-1GN inhibited the entry of both H5N1 and H1N1 influenza strains in the μM range by interacting with the hemagglutinin (HA) 2 subunit. This subunit is able to mediate the fusion with the host cell and, together with HA1, is responsible for the virus–receptor interaction and constitutes the HA glycoprotein.

Very similar activities are displayed by urumins (IPLRGAFINGRWDSQCHRFSNGAIACA) isolated from the Indian frog *Hydrophylax bahuvistara* [40]. The peptide exhibits an inhibitory effect against influenza A H1N1 virus [41] by targeting a very conserved region within the HA protein and disrupting the viral particle. Additionally, magainin 1 and 2 are AVPs found in amphibians, in particular from the frog *Xenopus laevis*. They inhibit herpes simplex virus type 1 (HSV-1) and 2 (HSV-2) infection [42], and an alanine-substituted magainin-2 amide is also reported to have antiviral potential against vaccinia virus [43]. This modified peptide was able to target the viral membrane via a carpet-based mechanism and completely destroy the viral surface. Dermaseptins constitute another important and large family of AVPs. They are isolated from the *Phyllomedusa* genus and their activity mainly targets HSV-1, HSV-2, and human immunodeficiency virus (HIV) [44,45,46]. In addition, the substitution of methionine in position four with lysine in dermaseptin S4 strongly improved the antiviral performance and also reduced peptide cytotoxicity. Recently, dermaseptins have also been tested for their anti-rabies virus activity and showed a strong effect in vitro when added 1 h after infection [47]. These findings evidenced a dual mechanism of action for dermaseptins, not only directed to the viral envelope but also in interfering with the intracellular infection of rabies virus.

Temporins are very short peptides (8–17 amino acid residues) that can be obtained from the *Ranidae* family, which has more than 1000 isoforms. Temporin G (FFPVIGRILNGIL-NH_2_) has been reported to prevent influenza A virus HA2 conformational changes that are essential for the fusion with the endocytic membrane, therefore inhibiting virus entry into the host cell [48]. The activity of temporin G was also retained against HPIV, in which the virus entry is arranged by a membrane fusion process. In this case, the peptide reduced the viral infection but probably not via a HA-targeting mechanism. Temporin L (FVQWFSKFLGRIL-NH_2_) is another member of the temporins family. It is characterized by a broad spectrum of action against different human pathogens, including both Gram-positive and Gram-negative bacteria, fungi, and viruses [49,50,51,52]. We have previously described its potential against SARS-CoV-2 and other coronaviruses, influenza virus, measles virus, and HPIV-3 [31], evidencing that the peptide and its analogues were able to interfere with the early stages of infection caused by enveloped viruses. In addition, we conjugated the best temporin L peptide analogue to cholesterol or fatty acids and observed an obvious reduction in cytotoxicity and an improvement in the antiviral performance. Recently we also reported the activity of three frog-derived peptides, namely AR-23, RV-23, and Hylin-a1, against a wide range of human and animal viruses [30,53]. In detail, AR-23, which is very similar to melittin in terms of the primary sequence, was active against a wide panel of human viruses, whereas Hylin-a1, derived from the skin secretion of the South American frog *Hypsiboas albopunctatus* (*H. albopunctatus*), showed a large-scale antiviral action against potential zoonotic viruses, including the canine distemper virus (CDV), a paramyxovirus affecting dogs and carnivores and responsible for a serious contagious infection of the respiratory tract.

Here, we continue to focus on Hylin-a1 by extending its spectrum of action in human respiratory viruses. The peptide exhibits a broad-spectrum antiviral activity against SARS-CoV-2, HCoV-229E, MeV, HPIV-3, RSV, and influenza virus. The target of the peptide is the viral envelope (present in all the analyzed viruses), which is irreparably damaged after treatment with the peptide. Our data show that Hylin-a1 could be used as a pan-inhibitor against different types of enveloped respiratory viruses. The discovery of an antiviral compound capable of acting on different types of viruses could be important to manage current and future pandemics.

## 2. Results

### 2.1. Hylin-a1 Was Not Cytotoxic towards Vero Cells

**Figure 1:**
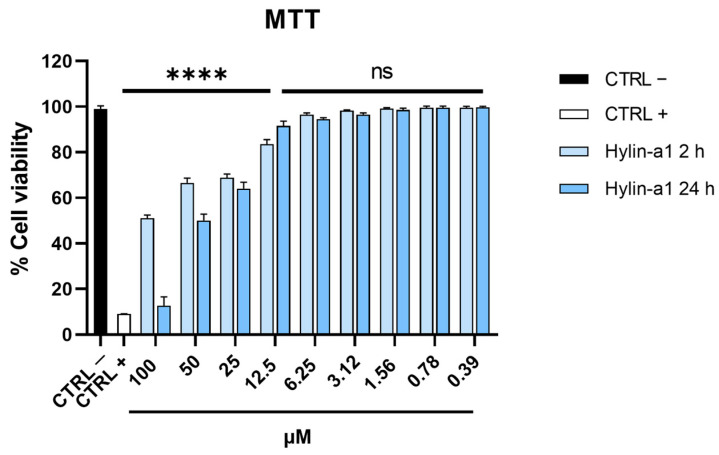
Hylin-a1 cytotoxic activity. Cells were treated with peptide at several concentrations ranging from 0.39 to 100 μM. After 2 and 24 h of treatment, cell viability was obtained by the spectrophotometer analysis of the MTT assay. Cells without peptide were used as a negative control (CTRL −), whereas DMSO was used as a positive control (CTRL +). **** *p* < 0.0001; ns: non-significant.

We first analyzed the cytotoxicity of the peptide in monkey kidney epithelial cells (Vero cells) in order to define the range of concentrations to use in the antiviral assays. The 3-(4,5-dimethylthiazol-2-yl)-2,5-diphenyl-2H-tetrazolium bromide (MTT) assay was employed to assess the mitochondrial ability to reduce MTT to formazan crystals, which is possible only in viable cells. Figure 1 shows that Hylin-a1 had a low cytotoxic effect after 2 h (h) of treatment at concentrations of 0.39–100 μM in this cell model, since the viability percentages of cells were ≥50% and similar to the control. On the other hand, when cells were treated with the peptide for 24 h, there was a dose-dependent toxic effect that reached a maximum at the highest concentration tested (100 μM).

### 2.2. Hylin-a1 Inhibited HCoV Infections

**Figure 2:**
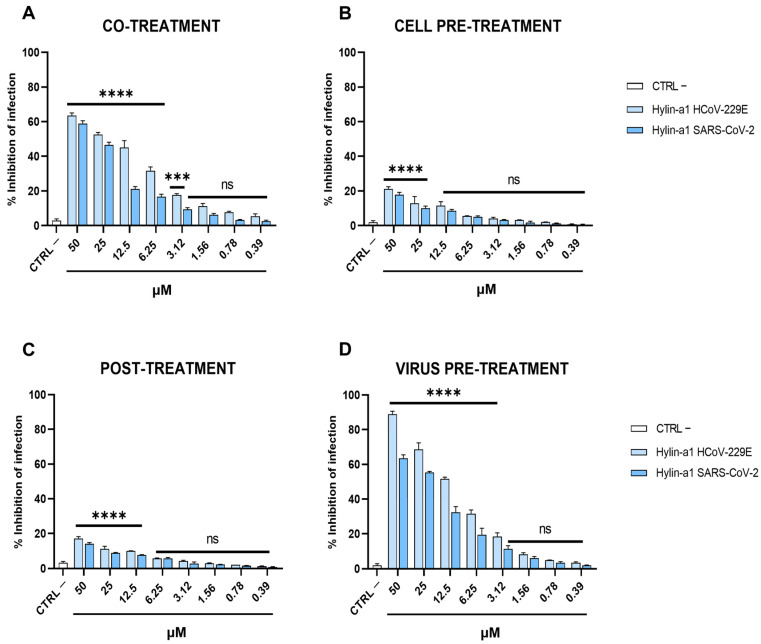
Antiviral activity against HCoV. Different assays (co-treatment, cell pre-treatment, post-treatment, and virus pre-treatment) were conducted in order to test the anti-HCoV activity. (**A**) Co-treatment; (**B**) cell pre-treatment; (**C**) post-treatment; (**D**) virus pre-treatment. Infected cells without peptide were used as controls (CTRL −). **** *p* < 0.0001; *** *p* < 0.001; ns: non-significant.

The peptide was assessed for its ability to inhibit the viral replication of HCoV-229E and SARS-CoV-2 in Vero cells. The cells were (i) exposed simultaneously to Hylin-a1 and each virus (co-treatment); (ii) treated with Hylin-a1 for 1 h before inoculation with HCoV (cell pre-treatment); (iii) exposed to Hylin-a1 after 1 h of infection (post-treatment); and (iv) treated with a pre-incubated mixture consisting of virus and Hylin-a1 (virus pre-treatment). The peptide was added in a range of non-cytotoxic concentrations from 0.39 to 50 μM, which exhibited different activities in several of the assays performed but was very similar in the two HCoVs used in the study. In the first assay, when cells were co-treated with the peptide and the HCoV, Hylin-a1 was able to inhibit infectivity in a dose-dependent manner (Figure 2A), with a 50% inhibitory concentration (IC50) at 12.5 and 25 μM for HCoV-229E and SARS-CoV-2, respectively. To better investigate the inhibitory effect of the peptide on coronavirus replication, we analyzed if Hylin-a1 directly affected viral particles or indirectly interacted with the pre-infected or post-infected host cells. The peptide did not show any activity in pre-infection (Figure 2B) or post-infection (Figure 2C) experimental conditions, suggesting that it did not have any prophylactic or curative actions, respectively. On the other hand, we observed an improvement in the antiviral activity in virus pre-treatment (Figure 2D), in which Hylin-a1 was active at similar relative concentrations against both viruses (IC50 values of 10 and 20 μM for HCoV-229E and SARS-CoV-2, respectively).

This result shows that Hylin-a1 probably inhibited the infection mainly by directly damaging the viral particles, thereby exhibiting a strong propensity for virucidal activity.

### 2.3. Hylin-a1 Inhibited Paramyxovirus Infection

**Figure 3:**
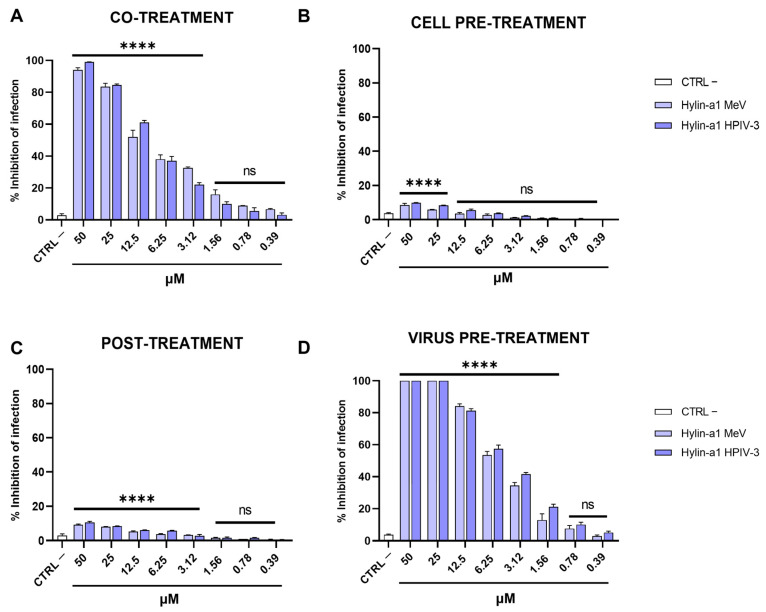
Antiviral activity against paramyxoviruses. Different assays were used to investigate the anti-MeV and anti-HPIV-3 activities. (**A**) Co-treatment; (**B**) cell pre-treatment; (**C**) post-treatment; (**D**) virus pre-treatment. Infected cells without peptide were used as controls (CTRL −). **** *p* < 0.0001; ns: non-significant.

To further define the inhibitory activity of Hylin-a1, in vitro antiviral assays were performed with a panel of paramyxoviruses, namely MeV, HPIV-3, and RSV. The same assays previously described were carried out for MeV and HPIV-3 (Figure 3), indicating the enhanced antiviral activity of the peptide. In detail, Hylin-a1 exhibited IC50 values of 12.5 and 8 μM for MeV and HPIV-3, respectively, when incubated together with the virus in the cell culture (co-treatment, Figure 3A). On the other hand, no activity was detected directly on the cell surface (cell pre-treatment, Figure 3B) or in any intracellular phase of infection (post-treatment, Figure 3C). Instead, we found that the virucidal activity of Hylin-a1 was notably increased in the virus pre-treatment assay (Figure 3D), with IC50 values of 6.25 and 5 μM for MeV and HPIV-3, respectively. We conclude from these experiments that the inhibitory effect occurred when the peptide interacted directly with the virus particle itself, thereby reducing the virus’ infectivity.

**Figure 4:**
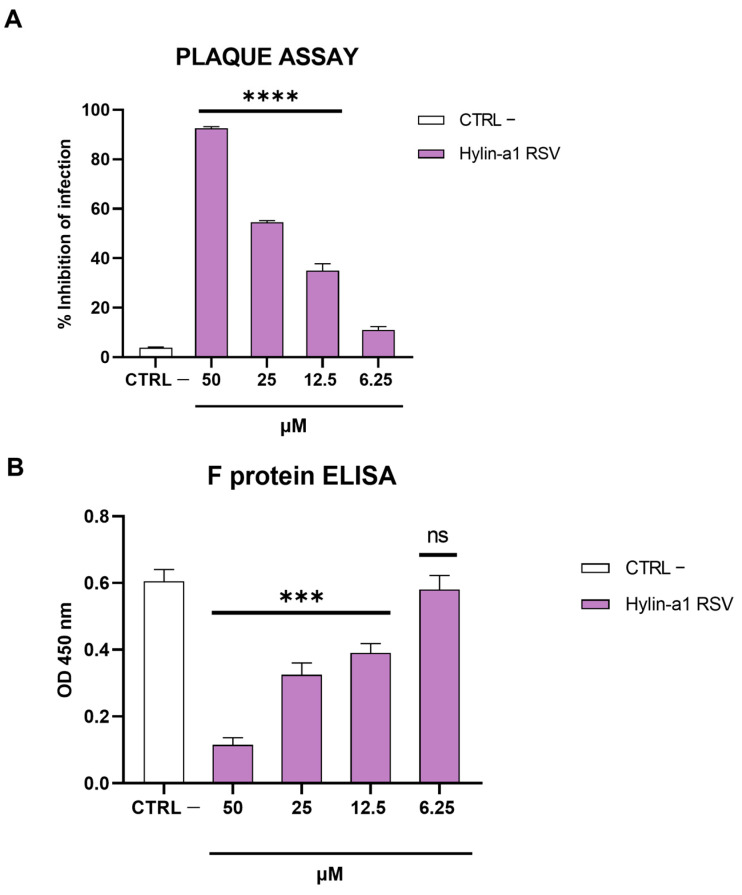
Antiviral activity against RSV. (**A**) Plaque assay. Co-treatment was performed and viral plaques observed after 48 h. Infected cells without peptide were used as controls (CTRL –). (**B**) RSV F protein ELISA. The binding of cells to the F protein was analyzed both in the absence and presence of Hylin-a1. **** *p* < 0.0001; *** *p* < 0.001; ns: non-significant.

To investigate if Hylin-a1 could also interfere with other paramyxoviruses, we analyzed the activity against RSV via a plaque assay and RSV fusion (F) protein enzyme-linked immunosorbent assay (ELISA). The cells were infected with RSV and treated in the presence or absence of Hylin-a1 within the range of 6.25–50 µM. For the plaque assay, Vero cells were used as model for RSV infection and viral plaques were observed after 48 h. On the other hand, at 20–24 h post-infection HeLa cells were assayed for the binding of the F protein. The results in Figure 4A show that Hylin-a1 was able to reduce RSV infection, albeit to a lesser extent than for other paramyxoviruses (Figure 3). Figure 4B shows a dose-dependent inhibition of the interaction with the surface protein when the peptide was incubated with RSV, suggesting that Hylin-a1 was able to block the membrane fusion and, subsequently, the viral infection.

### 2.4. Hylin-a1 Inhibited Influenza Virus Infection

**Figure 5:**
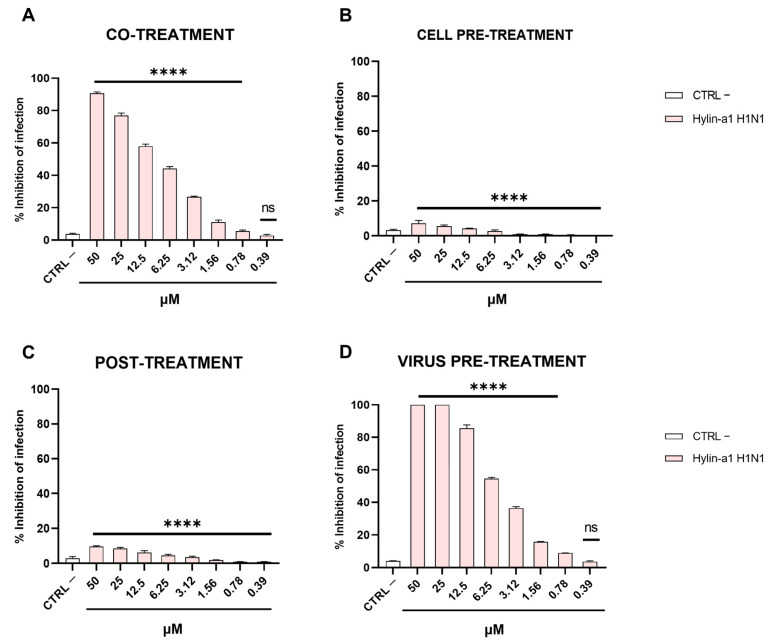
Antiviral activity against influenza virus. Different assays were carried out to verify the anti-influenza virus activity. (**A**) Co-treatment; (**B**) cell pre-treatment; (**C**) post-treatment; (**D**) virus pre-treatment. Infected cells without peptide were used as controls (CTRL –). **** *p* < 0.0001; ns: non-significant.

Once we had highlighted the virucidal activity of Hylin-a1 in HCoV-229E, SARS-CoV-2, MeV, HPIV-3, and RSV, we hypothesized that the compound could possess a broader virucidal activity against other viruses. Therefore, we assessed the effects of Hylin-a1 on the influenza H1N1 virus by performing the same set of experiments previously mentioned. We incubated the peptide and virus simultaneously with the cells and discovered a significant inhibitory effect against influenza virus in a concentration-dependent manner (Figure 5A). The IC50 value of Hylin-a1 in influenza virus was in the low micromolar range (10 μM). Next, we sought to determine if the peptide could act in other phases of influenza virus infection (Figure 5B–D). We confirmed its virucidal potential (Figure 5D). In addition, Hylin-a1 had no effect on the cell (Figure 5B) or inside the cell (Figure 5C). In detail, the IC50 value was 6.25 μM in the virus pre-treatment assay.

### 2.5. Hylin-a1 Did Not Inhibit Coxsackievirus Infection

Furthermore, we evaluated the inhibitory potential of Hylin-a1 against the nonenveloped coxsackievirus B3 (CVB3). The viral infectivity was not influenced by the presence of the peptide, as observed using plaque reduction assays following the four described experimental conditions (Supplementary Figure S1). These data indicate that Hylin-a1 is an inhibitory peptide that is specifically active on enveloped respiratory viruses.

## 3. Discussion

To date, the “one bug-one drug” approach has been successfully adopted in therapies against HIV, hepatitis C virus (HBV), and influenza virus infections [54,55]. However, this strategy has proved to be insufficient considering the huge variety of viral pathogens causing emerging and re-emerging infectious diseases. Therefore, the “one bug-one drug” theory has been recently replaced by the broad-spectrum antiviral agents (BSAAs) approach, which considers compounds sharing common characteristics capable of fighting two or more viruses [56]. Since the majority of pathogens are enveloped viruses, antivirals targeting the viral membrane constitute the most effective ones [57,58], as well as agents affecting similar pathways and host factors to replicate inside the cell [59]. In this study, we identified that the broad-spectrum antiviral peptide Hylin-a1 has potent antiviral activity against enveloped respiratory viruses, such as HCoV-229E, SARS-CoV-2, measles virus, HPIV-3, RSV, and influenza virus, whereas the peptide had no effect on the non-enveloped virus CVB3.

Since its isolation from the frog *H. albopunctatus*, Hylin-a1 has shown cytolytic properties against Gram-positive and Gram-negative bacteria and fungi [60]. Specifically, it was able to interfere with the growth of *Staphylococcus aureus* (*S. aureus*) (minimum inhibitory concentration (MIC) at 8 μM) and *Enterococcus faecalis* (MIC at 16 μM), whereas the peptide exhibited lower MIC values for *Escherichia coli* and *Pseudomonas aeruginosa* (MIC values at 32 μM and 64 μM, respectively). In addition, the same authors reported that Hylin-a1 exerted antifungal activities against *Candida* spp. (MIC at 16.7 μM for *Candida albicans* and *Candida krusei* and MIC at 67 μM for *Candida parapsilosis*). The antimicrobial activity of Hylin-a1 was highly influenced by the number of positive charges at the N-terminal end [61]. Crusca et al. observed a net improvement in the antimicrobial activity by adding a lysine residue at the N-terminal end, thus increasing the positive charge and the hydrophilic character of the peptide. On the other hand, the helicity of the acetylated peptide was reduced; therefore, there was a better correlation between the antimicrobial activity and, in particular, the antifungal activity and the hydrophilicity than for other parameters such as helicity. Other analogs of Hylin-a1 were recently designed to reduce toxicity and to maintain antimicrobial activity. In detail, Hylin-a1 K11 and Hylin-a1, carrying substitutions in lysine and alanine (I**AK**AILPLAL**K**ALKKLIK-NH_2_ and I**AK**AILPLAL**K**ALK**K**LIK-NH_2_, respectively), exhibited better stability and hemolytic profiles and had increased antibacterial and anti-biofilm activities against carbapenem-resistant *Acinetobacter baumannii* [62].

Despite the broad antibacterial activity of Hylin-a1, its antiviral potential has been poorly investigated. Very recently, we proved that Hylin-a1 was able to interfere with the infectivity of a series of animal viruses, namely CDV, Schmallenberg virus, bovine viral diarrhea virus, caprine herpesvirus, and bovine herpesvirus, which are characterized by a high potential to spill into humans [53]. This study is very interesting since the search for new agents able to eradicate the viral infection in animals is pivotal to overcome future human epidemics or pandemics due to emerging and re-emerging viruses. In the present study, we demonstrated that the same peptide could be considered a pan-inhibitor of human respiratory viral infections. The antiviral effect was targeted to the viral envelope in a nonspecific way, since the peptide was active against all the tested enveloped viruses. We hypothesize that the positive charges of the peptide and its amphipathic characteristic, rather than the composition of viral membrane, may allow Hylin-a1 to interact with the anionic phospholipids present on the viral surface, thereby damaging its architecture and curvature by some unknown mechanism and exerting virucidal activity. These data are also supported by our recent evidence regarding Hylin-a1 and its antibacterial activity [63]. We observed that the peptide exerted bactericidal action against *S. aureus* by destroying the bacterial cell wall. It is widely known that one of the mechanisms of action of AMPs involves membrane destruction, followed by bacterial lysis and death [64,65,66]. As the bacterial and viral membranes are very similar in lipid content, it is clear that Hylin-a1 may exert the same action by accumulating on the pathogen surface, causing membrane tension and pore formation.

We also demonstrated that the peptide interfered with RSV infection by targeting the fusion (F) protein. All viruses used in the present study are characterized by class I viral fusion proteins on their surfaces [67,68,69]. The fact that RSV F protein levels were drastically reduced after Hylin-a1 treatment indicated that the peptide could also affect the fusion machinery. Therefore, as well as a nonspecific action on the viral envelope, the peptide could also interact with specific viral proteins, suggesting a dual antiviral effect leading to virus inactivation.

In summary, Hylin-a1 could represent a pan-inhibitor against viral respiratory infections in an era in which emerging and re-emerging viral threats remind us of the urgent need for BSAAs.

## 4. Materials and Methods

### 4.1. Hylin-a1 Production

The peptide was synthesized and purified as reported elsewhere [58,70]. In detail, the peptide was synthesized as C-terminally amidated molecules following the Fmoc (N-9-fluorenylmethyloxycarbonyl) strategy and using oxyma/DIC and HA-TU/collidine as coupling agents, as reported in the literature [59]. The purity of the peptide was up to 98%.

### 4.2. Cells and Virus Culture

Vero cells (ATCC CCL-81, Manassas, VA, USA), Vero/hSLAM cells (ECACC 04091501, Porton Down, UK), Madin Darby canine kidney (MDCK, ATCC CCL-34), and HeLa cells (ATCC CCL-2) were cultured in Dulbecco’s Modified Eagle Medium (DMEM) with 4.5 g/L glucose (Microtech, Naples, Italy). Media were complemented with 100 IU/mL penicillin and 100 μg/mL streptomycin (Himedia, Naples, Italy) and 10% fetal bovine serum (FBS) (Microtech). MeV (ATCC VR-24) was propagated on VERO/hSLAM monolayers; RSV (ATCC VR-1540), HPIV-3 (ATCC VR-93), HCoV-229E (ATCC VR-740), SARS-CoV-2 (strain VR PV10734, kindly donated by the Lazzaro Spallanzani Hospital of Rome, Italy), and coxsackie type B3 (CVB3) strain Nancy (ATCC VR-30) were grown on Vero cells, whereas influenza virus (strain H1N1, VR-1894) was grown on the MDKC cell line, as reported elsewhere [31].

### 4.3. Cytotoxicity

The cytotoxic effect was analyzed on Vero CCL-81 cells in 96-well tissue culture plates (5 × 10^3^ cells/well) using the MTT (Sigma-Aldrich, St. Louis, MO, USA) assay [31]. Hylin-a1 was incubated at concentrations ranging from 100 to 0.39 µM for 2 and 24 h. At the end of incubation, 100 μL of MTT solution (5 mg/mL) was added and left on the cells for 3 h at 37 °C. Formazan salts were dissolved using 100 μL of 100% DMSO (Sigma-Aldrich) for 10 min with vigorous shaking; finally, cell viability was evaluated by spectrophotometer analysis at 540 nm. All experiments were performed in triplicate, and the means standard deviations are reported.

### 4.4. Antiviral Activity

Four different plaque assays were performed to evaluate the antiviral activity of Hylin-a1: (i) co-treatment; (ii) cell pre-treatment; (iii) post-treatment; and (iv) virus pre-treatment, where the main difference was the timing of the peptide addition [31]. Inhibition of infectivity percentage (% inhibition) was calculated by comparing the number of plaques obtained in the wells treated with the peptide with the plaques counted in the negative control (cells infected with virus but without peptide).

Co-treatment: cells were treated with Hylin-a1 (50–0.39 µM) and simultaneously infected with the virus at a multiplicity of infection (MOI) of 0.1 pfu/mL for 1 h at 37 °C. Cell pre-treatment: pre-cooled cells were treated with the peptide for 1 h at 4 °C. Each virus was then added (MOI 0.1 pfu/mL) for 1 h at 37 °C. Post-treatment: cells were first infected with virus (MOI 0.1 pfu/mL) for 2 h at 37 °C, after that, Hylin-a1 was incubated with the cells for another hour at 37 °C. Virus pre-treatment: the peptide was incubated together with the virus (1 × 10^4^ pfu/mL) for 1 h at 37 °C. The mixture of the virus/peptide was titrated onto cells to reach the virus MOI of 0.01 pfu/cell for 1 h at 37 °C.

At the end of each treatment, unabsorbed viruses were inactivated by citrate solution (pH 3) washing and cells were overlaid with carboxymethylcellulose at 5% (Sigma-Aldrich) supplemented with complete culture medium. When viral plaques were observed, cells were fixed with 4% formaldehyde (Sigma-Aldrich), stained with crystal violet (Sigma-Aldrich) solution 0.5%, and the number of plaques scored. Non-linear regression analysis using GraphPad Prism (version 8.0.1) was carried out to determine the IC50 values.

### 4.5. RSV F Protein ELISA Assay

HeLa cells were seeded in 12-well plates the day before the infection. When the cells reached 80% confluence, they were infected with RSV (MOI 1 pfu/mL) and treated at the same time with Hylin-a1 as in the co-treatment assay. At the end of viral adsorption stage, the HeLa cell monolayer was washed with citrate solution and supplemented with DMEM plus 10% FBS. After 72 h of incubation at 37 °C, the supernatants were collected and an ELISA assay was carried out as recommended by the kit guidelines (Sino Biological cod. KIT11049, Beijing, China).

### 4.6. Statistical Analysis

All experiments were performed in triplicate and expressed as mean ± standard deviation (SD) as calculated by GraphPad Prism software version 8.0.1. One-way ANOVA followed by Dunnett’s multiple comparisons test was performed; a value of *p* ≤ 0.05 was considered significant.

## Data Availability

The data presented in this study are available on request from the corresponding author. Authors can confirm that all relevant data are included in the article.

## Supplementary Materials

The following supporting information can be downloaded at: https://www.mdpi.com/article/10.3390/ijms241813888/s1.
