# Supplementary material for: Hylin-a1: A Pan-Inhibitor against Emerging and Re-Emerging Respiratory Viruses
Source: Int J Mol Sci. 2023 Sep 9;24(18):13888. doi: 10.3390/ijms241813888 (PMC10531407; doi:10.3390/ijms241813888)
Supplement: Supplementary file 1 [file ijms-24-13888-s001.zip › ijms-2555120-supplementary.pdf]

International Journal of *Molecular Sciences*

MDPI

Article

Hylin-a1: a pan-inhibitor against emerging and re-emerging respiratory viruses

Annalisa Chianese [1], Carla Zannella [1], Alessandra Monti [2], Nunzianna Doti [2], Giuseppina Sanna [3], Aldo Manzin [3], Anna De Filippis [1] and Massimiliano Galdiero [1,*]

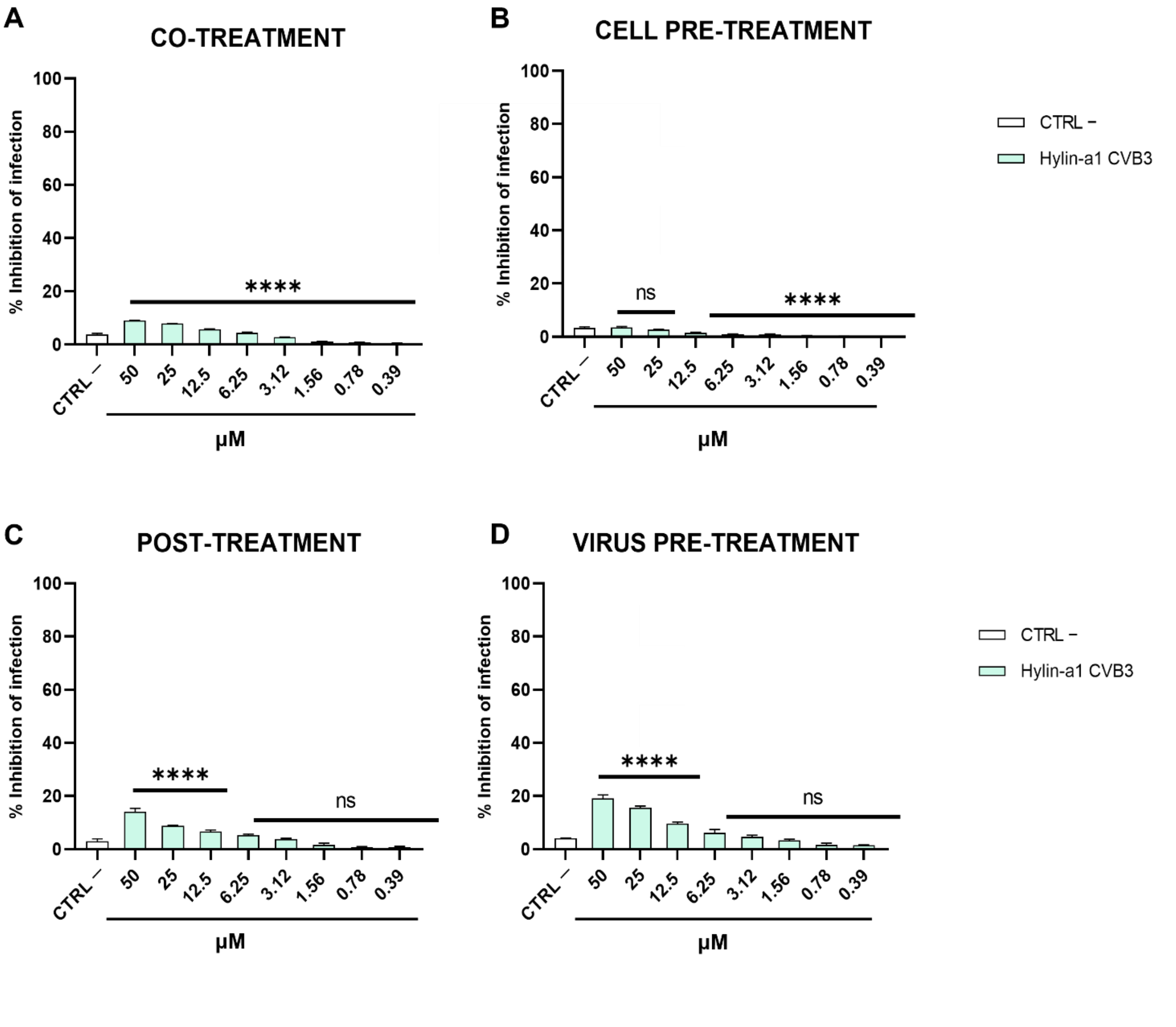

Figure S1. Antiviral activity against CVB3. Different assays were performed in order to evaluate antiviral activity. (A) co-treatment; (B) cell pre-treatment; (C) post-infection; (D) virus pre-treatment. Infected cells were used as negative control (CTRL -). ****p < 0.0001; ns: non-significant.
